# Case of Malignant Pustule

**Published:** 1884-06

**Authors:** Lionel A. Weatherly

**Affiliations:** Portishead


### CASE OF MALIGNANT PUSTULE. By LIONEL A. WEATHERLY, M.D., Portishead.

J. B., aged 18, a strong, healthy lad, came to see me on the morning of Friday, March 7th, about a slight swelling under the angle of his jaw, on the left side. He said he felt quite well in himself. In the centre of this swelling, which was more a simple puffiness, I noticed a small dark pustule about the size of half a threepenny-piece, round which were four small vesicles and an area of redness extending about a quarter of an inch, with a somewhat brawny feel on touching it. He said he had had a boil on his neck some few weeks before, which had discharged freely and got quite well; that he noticed the present pustule, which he called a "cat's-boil," on Tuesday, March 4th; that it had got the dark colour, and the vesicles had made their appearance, since Thursday, March 6th. He had been out of work (he was a dock labourer, and was sometimes employed in the sheds turning the grain) for some days, and had had nothing whatever to do among animals. On questioning him further on this point, I found that he had stood for some little time in a slaughter-house on the day previous, Thursday, March 6th, on which day it will be noticed that the dark colour and vesicles were first seen. He had had no rigor, and persisted in saying he felt quite well.

I sent him home at once, and ordered a large poultice to be applied. Within an hour I called at his house in order to make a free incision. In this short space of time the swelling had visibly increased. I made a free cross-incision through the pustule and hard brawny tissues, applied pure carbolic acid, four leeches in the neighbourhood of the pustule, and large poultices. I put him on a mixture of quinine and iron, and ordered him to have plenty of nourishment and stimulants.

On Saturday, March 8th, the swelling was still increasing down the front of his chest, down back and whole of the left side of his face. His constitutional symptoms were good; the leeches had taken a large quantity of blood; the pustule looked very dark, but showed no signs of sloughing; the vesicles had increased in size and number, but this was probably the result of the carbolic acid; his temperature was normal. On

Sunday, March 9th, I learnt he had passed a very restless night. The œdema had greatly increased in the neck, particularly in front, and so much so that from his difficulty in breathing I feared he might be suffocated from pressure on the trachea. Erysipelatous inflammation had set in, and reached as high as the eyebrow and as low as the clavicle. I painted a broad band of iodine outside the area of this, and ordered the same to be done night and morning. His pulse was feeble; but he complained of no pain, and said he did not feel very ill in himself. Temperature normal.

Monday, March 9th. Erysipelatous inflammation better, and swelling across front of the neck not quite so great; pustule looking very dark, and showing some sign of sloughing; area of brawniness increased. He had had a bad night, and was slightly delirious.

Tuesday, March 10th. I found him much worse. He had been very delirious through the night; the area of brawniness had again increased, and there was more evidence of sloughing. Mr. Greig Smith saw him with me at four o'clock, when he was in a semi-comatose state, and he died in this state at 7.30 p.m.

No *post mortem* examination was allowed, I am sorry to say. On Monday I sent some of the blood from the neighbourhood of the pustule to Dr. Shingleton Smith and Mr. Greig Smith, as they had kindly promised to examine it microscopically for me, and the result of their examination showed undoubted bacillus anthracis.

*Remarks.*—From the first moment I saw this case I felt that I had to deal with one of malignant pustule, and I venture to think that its result and the microscopical examination of the blood prove that I was right. I find that only two cases of this disease are recorded in the

Pathological Society's Report for last year—one by Mr. Davies Colley, and one by Mr. Bryant; but Mr. Colley mentions that they had had twenty-three cases during the past eleven years at Guy's, four of which had been received during the three months immediately preceding his paper.

In Mr. Colley's case the patient had been struck in the face and scratched by a hide; he had become restless, and had had a rigor. On admission the man was very ill in himself, and there was a bright red swelling on the cheek, with an abrupt edge and a dark slough in the centre. The tumour was at once excised, and the patient got quite well.

In Mr. Bryant's case the tumour was also excised, but the patient died collapsed in ten hours.

In both these cases bacilli were found in the serum of the tumours, in the urine, fæces, and sweat; but Mr. Colley makes a point of the fact that micrococci only were found in the blood. In Mr. Colley's case there was no distinct ring of vesicles, and the constitutional symptoms came on early.

With regard to my case, the peculiarities seem to be the following:—(1) One cannot trace the cause definitely, although one may fairly surmise that he might have had an ordinary small boil up till Thursday, which might in some way have been inoculated during his short stay in the slaughter-house, where there were hides of animals lying. (2) The absence of rigor, and the lateness of distinct constitutional symptoms. (3) The absence of the usual prominent condition of sloughing.

It is much to be regretted that no *post mortem* examination could be made in this case. It would have been interesting to have examined the internal organs, and seen whether or not they were affected by the pustule, as in the majority of such cases; and after reading Mr. Colley's case I cannot but wish that I had at once excised the tumour, but I thought that free incision and the application of the pure carbolic acid would have sufficed.

